# Microsurgical techniques in the treatment of breast cancer-related lymphedema: a systematic review of efficacy and patient outcomes

**DOI:** 10.1007/s12282-021-01274-5

**Published:** 2021-07-12

**Authors:** Konstantinos Gasteratos, Antonios Morsi-Yeroyannis, Nikolaos Ch. Vlachopoulos, Georgia-Alexandra Spyropoulou, Gabriel Del Corral, Kongkrit Chaiyasate

**Affiliations:** 1 Department of Plastic and Reconstructive Surgery, Papageorgiou General Hospital, Thessaloniki, Greece; 2 Department of General Surgery, Hippokrateio General Hospital of Thessaloniki, Thessaloniki, Greece; 3 251 Hellenic Air Force General Hospital, Athens, Greece; 4 Department of Plastic and Reconstructive Surgery, Medstar Georgetown University Hospital, Washington, DC USA; 5 Division of Plastic and Reconstructive Surgery, Oakland University William Beaumont School of Medicine, William Beaumont and Beaumont Children’s Hospital, 3555 W 13 Mile Rd, Suite N120, Royal Oak, MI 48073 USA

**Keywords:** Breast cancer lymphedema, Postoperative complications, Microsurgery, Microsurgical procedures, Transplantation, Autologous, Lymphaticovenous anastomosis (LVA), Vascularized lymph node transfer (VLNT)

## Abstract

**Introduction:**

Secondary lymphedema is the abnormal collection of lymphatic fluid within subcutaneous structures. Patients with lymphedema suffer a low quality of life. In our study, we aim to provide a systematic review of the current data on patient outcomes regarding breast cancer-related lymphedema (BCRL), and the most prevalent reconstructive techniques.

**Methods:**

A PubMed (MEDLINE) and Scopus literature search was performed in September 2020. Studies were screened based on inclusion/exclusion criteria. The protocol was registered at the International Prospective Register of Systematic Reviews (PROSPERO), and it was reported in line with the PRISMA statement (Preferred Reporting Items for Systematic Reviews and Meta-Analyses).

**Results:**

The search yielded 254 papers from 2010 to 2020. 67 were included in our study. Lymphaticovenous anastomosis (LVA)—a minimally invasive procedure diverting the lymph into the dermal venous drainage system—combined with postoperative bandaging and compression garments yields superior results with minimal donor site lymphedema morbidity. Vascularized lymph node transfer (VLNT)—another microsurgical technique, often combined with autologous free flap breast reconstruction—improves lymphedema and brachial plexus neuropathies, and reduces the risk of cellulitis. The combination of LVA and VLNT or with other methods maximizes their effectiveness. Vascularized lymph vessel transfer (VLVT) consists of harvesting certain lymph vessels, sparing the donor site’s lymph nodes.

**Conclusion:**

Together with integrated lymphedema therapy, proper staging, and appropriate selection of procedure, safe and efficient surgical techniques can be beneficial to many patients with BCRL.

## Introduction

### Breast cancer-related lymphedema (BCRL)

Lymphedema can be defined as the abnormal collection of lymphatic fluid within subcutaneous structures. The causes are mostly iatrogenic, such as axillary lymph node dissection (ALND) and radiotherapy. Upper extremity lymphedema presents usually secondary to the treatment of breast cancer. Subjective or self-reported complaints of arm swelling or heaviness and/or an interlimb volume difference of > 200 mL (or > 10% [1]) are considered elements of BCRL [2].

According to literature, BCRL incidence depends on the treatment: axillary lymph node dissection results in lymphedema in up to 53.5% of cases and sentinel lymph node biopsy in up to 15.8% of cases [3, 4]. Most BCRL cases occur late post primary breast reconstruction procedures; thus, patient follow-up may affect the measured incidence rates and lead to varying results [5]. Risk factors for developing lymphedema include adjuvant radiation, docetaxel chemotherapy, infection, iatrogenic injury, and obesity [6–12]. Brunelle et al. reported that cording (also known as axillary web syndrome or Mondor’s disease) is an independent risk factor for BCRL, and it should be part of the lymphedema risk stratification protocol [13]. In a large Swedish breast cancer registry with over 57,000 women, lymphedema was amongst the conditions with highest hazard ratio for patient morbidity [14].

Breast cancer-related lymphedema symptoms negatively impact patients’ quality of life (QoL) [2, 15]. Depending on the severity of symptoms and imaging techniques data, the stage of lymphedema is deduced and classified according to the International Society of Lymphology (ISL)[16] (Table 1) or other staging systems that take into account different parameters, such as Campisi’s grading scale (Table 2) [17] and accordingly propose either conservative or surgical treatment scale, such as the Cheng’s grading scale (Table 3) [18]. Lymphoscintigraphy (LG), indocyanine green (ICG) and magnetic resonance lymphography (MRL) are the main imaging techniques used to outline the functional status of the lymphatic system [7, 15, 19, 20]. Functional lymphatics present linear flow, in contrast to compromised areas where dermal backflow is visualized and is further classified according to severity as seen in Table 4 [7, 9, 21]. Another imaging technique that may help understanding of underlying lymphatic pathophysiology and selection of therapeutic options is ultrasonography [22].

**Table 1:** ISL stages for classification of a lymphedematous limb [16]

Stage	Symptoms
0	Latent or subclinical lymphedema
I	Lymphedema which subsides with limb elevation; pitting may occur
II	Lymphedema rarely subsides with limb elevation alone; pitting is manifested
II (late)	Pitting edema is not present as excess subcutaneous fat and fibrosis develop
III	Lymphostatic elephantiasis; pitting may be absent; acanthosis, dermal thickening, further deposition of fat, tissue fibrosis, and warty overgrowths may develop

It should be noted that more than one stage could be identified in a limb

**Table 2:** Campisi’s clinical lymphedema staging [17]

Stage	Symptoms
1A	No edema despite the presence of lymphatic dysfunction
1B	Mild edema which subsides with limb elevation and night rest
2	Persistent edema which subsides only partially with limb elevation and night rest
3	Persistent, progressive edema; recurrent acute erysipeloid lymphangitis
4	Fibrotic lymphedema with column limb
5	Lymphostatic elephantiasis with severe limb deformation; column limb; scleroindurative pachydermatis, warty overgrowths may develop

**Table 3:** Cheng lymphedema grading [18]

Grade	Symptoms	Circumferential difference	Lymphoscintigraphy	Management
0	Reversible	< 9%	Partial occlusion	CDT
I	Mild	10–19%	Partial occlusion	LVA, liposuction, rehabilitation
II	Moderate	20–29%	Total occlusion	VLNT, LVA
III	Severe	30–39%	Total occlusion	VLNT + additional procedures
IV	Very severe	> 40%	Total occlusion	Charles procedure + VLNT

*LVA* lymphaticovenous anastomosis, *VLNT* vascularized lymph node transfer

**Table 4:** ICG lymphography classification of the functional status of the lymphatic system [7, 9, 21]

Lymphatic system’s status	ICG flow/pattern
Functional	Linear
Semi-functional	Dermal backflow
	Splash
	Stardust
	Diffuse
Non-functional	No flow

*ICG* indocyanine green

Microsurgical lymphatic surgeries are largely classified into “lymphatic bypass” and “lymphatic transfer”. “Lymphatic bypass” includes lymphaticolymphatic bypass (LLB) [23–26] and lymphovenous bypass (LVB) [27]. LVB is further classified into lymph node-to-venous shunt (LNVS) [28], lymphaticovenous implantation (LVI) [29–31], and lymphaticovenular anastomosis; LVI is also known as lymphatic microsurgery, classical lymphaticovenous anastomosis (LVA) [32–34], or telescopic lymphovenous anastomosis, where lymph vessels are inserted into a relatively large vein (lymphatic microsurgical preventive healing approach, LYMPHA is also included here). Lymphaticovenular anastomosis is also called as lymphatic supermicrosurgery or supermicrosurgical LVA, where a lymph vessel is anastomosed to a venule or small vein in an intima-to-intima coaptation manner. “Lymphatic transfer” includes vascularized lymph node transfer (VLNT) [35, 36] and vascularized lymph vessel transfer (VLVT) [37, 38]. This categorization is critical from the lymphological point of view.

In this study, we present a systematic review of the current physiologic microsurgical procedures that aim to restore a functional lymphatic drainage system, their indications, their efficacy and their impact on patients’ QoL. The postoperative outcomes are measured by a variety of modalities: physical exam, photography, circumference measurements (pre- and postoperative relative volume change [39]), computerized tomography (CT) scans, lymphoscintigraphy, and Lymphedema Quality of Life (LYMQOL) questionnaire (function, appearance, symptoms, mood) [40].

## Materials and methods

This review was registered at the International Prospective Register of Systematic Reviews on 18 September 2020 (PROSPERO, CRD42020157010) of the National Institute for Health Research, and it is reported in line with the Preferred Reporting Items for Systematic Reviews and Meta-Analysis (PRISMA) guidelines.

### Literature search

An electronic literature search was performed in PubMed (MEDLINE) and Cochrane database in September 2020. Basic keywords used in the search string were “microvascular”, “microsurg*”, “breast”, “lymph”, “lymphedema” in a combination of Boolean operators (Table 5). Filters were applied: full text, English, within 10 years, Humans, Female, and Adults.

**Table 5:** Complete search strategy for the systematic review

Keywords	#1 lymphedema #2 breast #3 surgery OR microvascular OR microsurg*	
#1 AND #2 AND #3 + Title/Abstract filter	((lymphedema[Title/Abstract]) AND (breast[Title/Abstract])) AND (((surgery[Title/Abstract]) OR (microvascular[Title/Abstract]) OR (microsurg*[Title/Abstract])))	
Search results	PubMed (MEDLINE): 845	Cochrane (Embase filter): 5 Clinical Trials

### Study selection (inclusion/exclusion criteria)

Only studies following the predetermined criteria were included. Studies should concern adult patients with secondary upper limb lymphedema due to breast cancer, undergoing microsurgical reconstruction to reduce lymphedema. If lower limb lymphedema, gynecological (cervical, uterine) cancer, or melanoma were the main topics, these studies were excluded from the review. Preventive microsurgical techniques and pharmacotherapy agents, conservative methods (e.g., manual lymphatic drainage, exercises) were also excluded, as well as microsurgical experimental techniques on animal models. Low-level evidence (i.e., case reports, letters to the editor) were excluded.

### Primary and secondary outcomes

Primary outcomes are patient centered. The primary outcome was the microsurgical techniques and the possible combinations available to the surgeon. The secondary outcomes were the efficacy of the microsurgical techniques and the complications that might occur.

### Data extraction

**Fig. 1:**
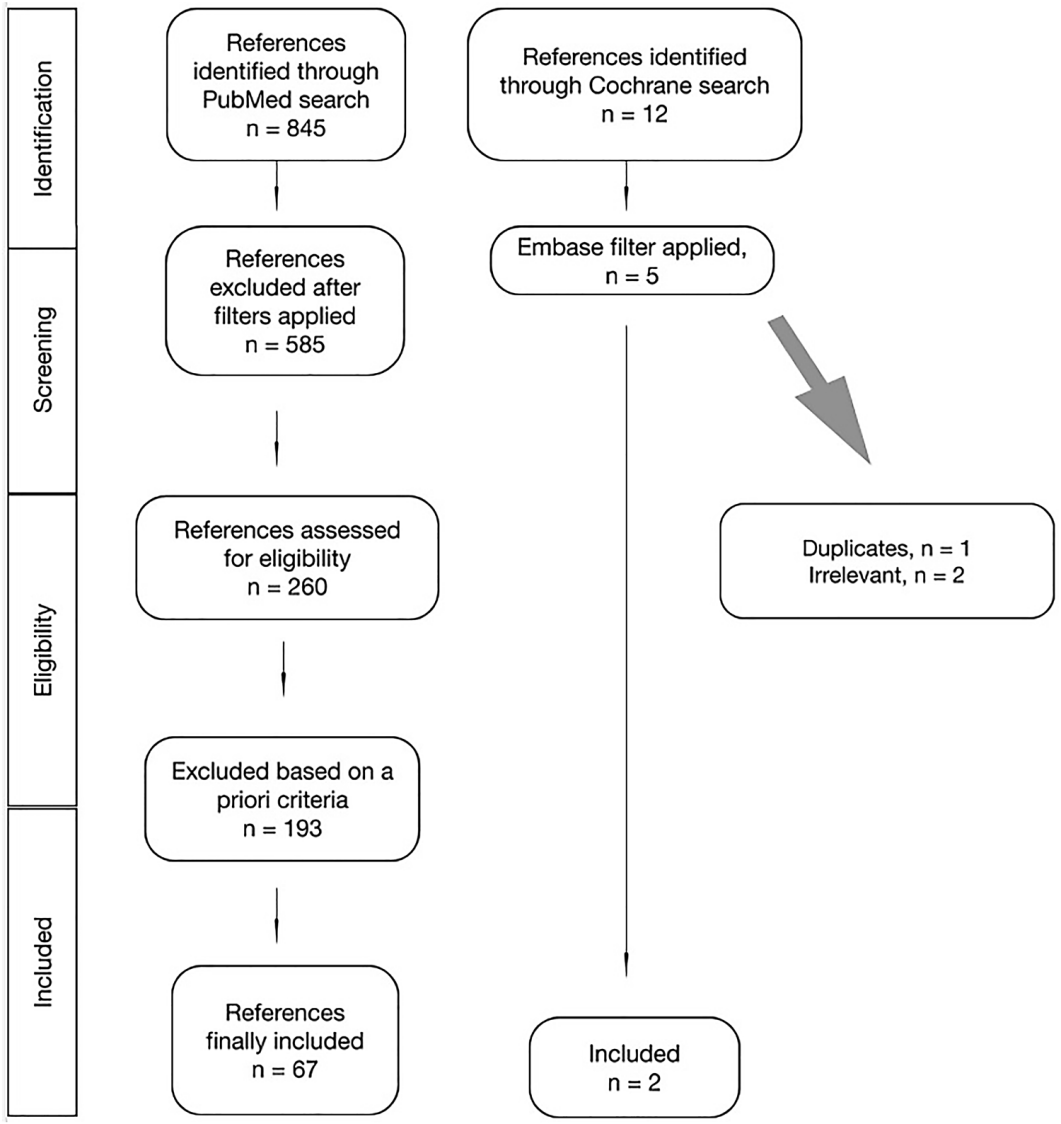
PRISMA flowchart of our systematic review

All search results were imported to the Covidence screening tool. After duplicate removal, two reviewers (KG, AM-Y) independently screened all titles and abstracts with full texts for eligible studies. Any discordance regarding study eligibility was resolved by consensus. The PRISMA flowchart is shown in Fig. 1.

## Results

PubMed search yielded 845 results. The application of filters (full text, English, within 10 years, Humans, Female, Adults) generated 260 results out of which 67 were included in our study. Cochrane search yielded five clinical trials (Embase filter applied) out of which only two were relevant. A total of 69 papers were included in our study. A summary of these studies is listed in Table 6.

**Table 6:** Summary of major studies on BCRL microvascular treatments

Authors (year)	Type of study	Patients (*n*)	Mean age (years)	Lymphedema stage	Follow-up (months)	Intervention	Outcome	Conclusion
Jørgensen et al. 2018 [8]	Systematic review and meta-analysis	270	N/A	N/A	− 69	LYMPHA	Statistically significant reduction in lymphedema incidence	More studies are required to prove the efficacy of prophylactic LVA
Feldman et al. 2015 [64]	Prospective	24	Mean: 58.1 ± 11.8; range: 33–76	0	Mean: 3; range: 3–24	LYMPHA	Transient lymphedema reduction to 12.5%	LYMPHA early results are promising as a preventive technique for BCRL
Boccardo et al. 2011 [60]	RCT	46	67	N/A	18	LYMPHA (treatment group)	Significant difference between the two groups in the volume changes	No statistically significant differences in the arm volume in treatment group
Hahamoff et al. 2019 [65]	Retrospective	87	60	N/A	22	LYMPHA	LYMPHA with ALND decreased rate of lymphedema from 40% to 12.5%	Long-term follow-up and RCTs needed
Chang 2010 [27]	Prospective	20	54	Campisi stage II: 10; Campisi stage III: 10	18	LVA	95% symptom improvement, mean volume differential reduction	LVA effectively improves BCRL
Damstra et al. 2009 [43]	Prospective	10	58.7	Campisi stage III	24	LVA	2% volume reduction 1 year postoperatively	LVA in BCRL patients results in minimal improvements. Conservative is the treatment of choice for early stages BCRL
Chang et al. 2013 [42]	Prospective	89	54	Any	Mean: 30.4; range: 3–84	LVA	96% reported symptom improvement, 42% volume reduction at 12 months	LVA effectively improves early staged BCRL, ICG lymphography advantageous for lymphedema staging and treatment selection
Winters et al. 2019 [46]	Retrospective chart review	12	58.5	Campisi stage: I–IIa	12	LVA	32.3% decrease in arm volume difference, quality of life increased	At least 56.5% of LVAs patent after 1-year follow-up
Winters et al. 2017 [47]		29	57	Campisi stage: Ib–IIa	6 and 12	LVA	Statistically significant decrease in mean difference in arm volume and symptoms score	Significant improvement in QoL
Poumellec et al. 2017 [48]	Retrospective	31	64	Campisi stage: II–IV	12.8	LVA	Reduction in arm circumference, moderate to substantial functional improvement	Encouraging results, but poor LVA outcomes in Campisi stage 3 and 4 patients
Koshima et al. 2000 [32]	Prospective	27	LVA group: 57; CDT group: 62	N/A	LVA group: mean: 2.2, range: 1–72; CDT group: N/A	LVA; CDT	Average decrease in arm circumference 4.1 cm	LVA followed by CDT should be considered for lymphedema treatment
Mulken et al. 2020 [74]	RCT (pilot)	20	60 ± 11 (60 ± 7)	ISL classification Stage 1–2b	1 and 3	Robotic LVA (treatment group)	Improved QoL, decrease in arm volume Longer robot-assisted duration to complete LVA, worse anastomosis scores	Promising technique
Becker et al. 2012 [5]	Review	N/A	N/A	N/A	Minimum: 36	VLNT	VLNT is an effective treatment for secondary lymphedema, VEGF-C factor improves lymphangiogenesis	Breast reconstruction can be addressed simultaneously with VLNT, brachial plexus neuropathies improved

*N/A* not applicable, *RCT* randomized controlled trial, *ISL* International Society of Lymphology, *LYMPHA* lymphatic microsurgical preventive healing approach, *QoL* quality of life, *ICG* indocyanine green, *CDT* complete decongestive therapy, *LVA* lymphaticovenous anastomosis, *VLNT* vascularized lymph node transfer, *BCRL* breast cancer-related lymphedema, *ALND* axillary lymph node dissection, *VEGF-C* vascular endothelial growth factor C

The study designs and treatment methods used were highly variable among the articles. The data retrieved were heterogeneous and could not be combined numerically. Therefore, a systematic review was performed without a meta-analysis. After careful investigation of the current bibliography, two microsurgical techniques were more prevalent: lymphaticovenous anastomosis (LVA) and vascularized lymph node transfer (VLNT).

### Lymphaticovenous anastomosis (LVA)

#### Indications, imaging, and surgical technique

Lymphaticovenous or lymphovenous anastomosis (LVA) is indicated when the patient still has a functional lymphatic system, but with underlying blockage causing lymphedema, and a venous system with intact valves to avoid venous-lymphatic reflux [7, 15]. The principle of this approach is to divert the lymph into the subdermal venous drainage system. This procedure can be done either under general or local anesthesia due to its minimally invasive nature [7, 15, 41]. When the LVA is performed right after the axillary lymph node dissection, it is known as LYMPHA, or “immediate lymphatic reconstruction”.

The lymphatic channels are usually outlined with distally injected blue dye and ICG lymphography [3, 4, 8, 15, 42]. If any of these modalities do not visualize functional lymphatics, other technologies can be used such as ultrasound and MRL [7].

Usual lymphatic vessel diameter is around 0.1–0.6 mm [3, 43]. An important technical pearl is that the diameter of the subdermal venules should be less than 0.8 mm, and anastomosed in an intima-to-intima manner to avoid venous-lymphatic reflux and thrombosis seen with larger veins [8, 44]. Prior to anastomosis, the venule is transected and checked for venous backflow. If significant backflow is seen, a different vein is chosen. There are four types of anastomosis that can be performed: (a) end-to-end; (b) side-to-end; (c) side-to-side; (d) end-to-side [7]. According to Yamamoto et al. side-to-end and side-to-side types achieve the best drainage outcome, while end-to-side more often results in venous-lymphatic reflux and thrombosis [45]. Regarding the location and number of anastomoses, one or more can be performed in one or multiple levels of the affected extremity (e.g., wrist, forearm, and arm) [15]. Therefore, LVA can be characterized as orthotopic or heterotopic according to whether it is performed at the anatomical site of the lymphadenectomy or another site, and as immediate or delayed according to the time it is performed [8]. Post-operatively, the patency of the LVA anastomoses can be ensured with the same imaging techniques used in the preoperative staging of the disease [15]. It is estimated that at least 56.5% of the LVA anastomoses were patent at 1-year follow-up [46].

#### Outcomes

The prospective study by Damstra et al. demonstrated that LVA in patients with advanced chronic BCRL does not result in significant limb volume reduction and QoL improvement. Yet, a short initial period of symptoms alleviation was observed in some cases [43]. Similarly, the prospective study by Chang et al. reported an immediate improvement of symptoms which lasted for a short period of time. However, not all cases were accompanied with quantitatively measurable improvement, perhaps due to chronic fibrosis [27]. Winters et al. reported 29 women with unilateral BCRL who underwent LVA; arm volume was reduced and the QoL significantly improved [47]. A prospective study by the same author with 100 patients demonstrated a mean volume reduction of 61% in early-stage upper extremity lymphedema and a mean volume reduction of 17% in advanced stages of lymphedema [42]. The study by Koshima et al. showed that patients who underwent LVA and continued complete decongestive therapy (CDT) afterwards had 4.1 cm arm circumference reduction 2 years postoperatively, in contrast to the group that received CDT alone and observed a 75% reduction [32].

A general consensus on the optimal technique of LVA is yet to be devised in the medical community (number, levels, configuration of anastomoses), but the majority agrees that the earlier it is performed, the better the outcome [7, 8, 15, 42]. In addition, it is not considered as a cure, but rather as a means to reduce the severity of lymphedema’s symptoms in patients [48]. Therefore, to preserve the improvements gained after LVA, CDT should be continued after LVA, such as compression sleeves as per Chang’s et al. suggestion [27, 32, 49]. Immediate limb compression can enhance LVA results [50]. Possible complications can be venous-lymphatic reflux, thrombosis, infections, lymphatic fistula and wound healing problems [8, 15]. If the lymphedema’s stage advances despite LVA, the next treatment option would be a VLNT [7].

### Vascularized lymph node transfer (VLNT)

#### Pathophysiology

Vascularized lymph node transfer (VLNT) or autologous lymph node transplant (ALNT) is a microsurgical treatment option for lymphedema. It brings vascularized tissue into the previously operated field, but also near the wrist joint and elbow joint, such as Ming-Huei Cheng's submental node transfer [30, 36]. One theory suggests that, after anastomosing the arterial and venous vessels of recipient and donor sites, vascular endothelial growth factors (VEGFs) are released. These promote lymphangiogenesis, bridging the distal (occluded) with the proximal (healthy) lymph channels, and the development of new lymphovenous communications [5, 7, 15, 51]. Lymph nodes with important immunologic function are also brought into the fibrotic and damaged tissue [5]. Jung-Ju Huang et al. showed that lymph node transfer improves T cell-mediated immune responses in an experimental transgenic mouse model of lymphedema. Adequate levels of T and B cells are produced with an equal transport capacity after 30 days [52]. Another theory is that the placement of a vascularized lymph node may act as a “pump” to absorb fluid and redirect it into the vascular network [7, 15, 53]. Lastly, the prospective study by Garcés et al. showed that except from new connections of the lymphatic vessels with the venous system, intra tissue communications develop as demonstrated by ICG lymphography, increasing the lymph drainage [54].

The groin flap is the most frequently harvested lymph node flap [7, 15]. It is preferred because the scar is hidden, multiple lymph nodes can be harvested in the same flap, it has reliable anatomy and it can be combined with a deep inferior epigastric artery perforator (DIEP) flap for autologous breast reconstruction [5, 15, 55]. Other options for VLNT are the submental, supraclavicular, omental/gastroepiploic, thoracic and jejunal lymph nodes flaps, presenting different advantages or disadvantages (Table 7) [15].

**Table 7:** Lymph node flaps for VLNT [15]

Lymph node flap	Perfusion	Advantages	Disadvantages	Combinations	DSL risk
Groin	Superficial circumflex iliac vessel	Hidden scar; multiple lymph nodes; reliable anatomy; skin abundance	Short vascular pedicle	Simultaneous abdominally based breast reconstruction	Great
Submental	Submental artery/facial artery	Multiple lymph nodes; reliable anatomy; accessibility; great size of facial artery; flap thickness	Short vascular pedicle leading to facial vessels inclusion; risk for marginal mandibular nerve injury; platysma palsy; visible scar		Low
Supraclavicular	Transverse cervical artery	Hidden scar; complex anatomy	Small flap size; carotid artery injury; internal jugular vein injury; thoracic duct injury; phrenic nerve injury; supraclavicular nerve injury		Low
Omental/gastroepiploic	Right gastroepiploic artery	Hidden scar; multiple lymph nodes; reliable anatomy	Absence of cutaneous component for coverage; peritoneal entry and abdominal complications		Low
Thoracic	Lateral thoracic or thoracodorsal artery	Hidden scar; multiple lymph nodes; accessibility; long vascular pedicle	Thoracodorsal nerve loss	Scar removal surgeries and perforator flap	Great
Jejunal	Mesenteric vessels	Hidden scar; multiple lymph nodes; reliable anatomy	Absence of cutaneous component for coverage; peritoneal entry and abdominal complications	Simultaneous abdominally based breast reconstruction	Low

*DSL* donor site lymphedema

#### Indications and imaging

The indication for VLNT is complete blockage (Cheng’s stage II and above), loss of lymph nodes as seen by the absent signal in the LG and MRL, failure of conservative treatment and CDT, recurrent episodes of cellulitis, and brachial plexus neuropathies [15]. This procedure is best suited for patients who have identifiable active lymphatic channels as seen with ICG scanner, such as a splashback pattern [5].

To safely harvest a vascularized inguinal lymph node flap, axillary reverse mapping (ARM) should be performed in the donor area, using ICG, LG, lymphazurin, isosulfan blue or methylene blue dye in the knee and foot [15, 51]. This method allows for maximal preservation of lymphatic vessels [56, 57]. Then, during VLNT, the lymphatics other that the sentinel lymph nodes should be selected for surgical removal to reduce the risk of iatrogenic donor site lymphedema (DSL) [5, 51]. Intraoperative LASER mapping of donor sites using ICG, technetium tracer or LG can be used to assist in the selection of a lymph node flap [3, 7, 15, 51].

#### Surgical technique

Inset of the lymph node flap can be performed over the axillary vein, where lymphatic tissue was originally resected for cancer treatment. Concomitantly, scar from previous operations is removed. Theoretically, the edema will dissipate through lymphangiogenesis improving the overall cosmetic outcome. Other sites are the elbow or wrist; these distal sites can be selected to better place the harvested lymph nodes according to the lymphedema’s level and increase their “pump” effect, while another reason is the lack of difficulty of recipient bed dissection in contrast to the axilla. The cosmetic outcome of this approach may be addressed in subsequent operations, by removing the excessive skin, while preserving the benefits of the VLNT [15].

Nowadays, lymphedema and breast reconstruction can be addressed in a single operation. That being said, VLNT can be done simultaneously with microsurgical autologous breast reconstruction using a DIEP flap or a muscle-sparing transverse rectus abdominis myocutaneous (MS-TRAM) flap coupled with lymph nodes from the groin harvested along with the superficial circumflex iliac or superficial inferior epigastric vessels [3, 5, 51]. Another technique consists of using a latissimus dorsi (LD) flap coupled with the lateral thoracic lymph nodes [51]. Alternatively, a non-abdominal free flap, such as the thoracodorsal artery perforator (TDAP) flap combined with an inguinal lymph node flap can be used [5].

#### Outcomes

According to researchers, lymphedema might be greatly reduced, when LVA and VLNT procedures are used in combination [51]. Similarly, others report that improvements can be anticipated in the immediate postoperative period, perhaps due to the release of scar tissue of the previously operated and/or radiated field that may have been blocking the lymphatic flow [3, 5, 58]. Moreover, the study of Becker et al. reported that 40% of their patients with ISL stage I and II lymphedema did not require CDT after VLNT, while 95% of the ISL stage III lymphedema patients still had to continue CDT, despite some signs of improvement [5, 7].

Recently liposuction procedures have been used at the time of VLNT or in second stage [5]. Some researchers, such as Granzow et al. reported the combination of suction-assisted protein lipectomy (SAPL) and VLNT procedures in the treatment of chronic solid-phase lymphedema. SAPL was preceded by removing the solid materials and reducing the limbs circumference, and at a later stage VLNT was performed to improve lymphatic drainage and prevent fluid re-accumulation. The outcome of this approach reached volume reductions of over 83%, but CDT was still needed in the evenings and at night according to their findings. Notably by undergoing SAPL, without addressing the pathophysiology causing lymphedema, the incidence of severe episodes of cellulitis could be diminished by 75% or more [3].

The possible complications that may occur are thrombosis, seroma, lymphocele, hematoma, delayed wound healing and DSL among others [3, 5, 15, 51].

## Discussion

Five prospective studies, one systematic review and meta-analysis and one literature review were included for assessing the efficacy of LVA and VLNT procedures, as well as the QoL postoperatively (Table 6). Two were centered on LYMPHA, four on LVA and one on VLNT procedure. Three studies were published from 2000 to 2009, while the rest, during the last decade. All studies agreed that BCRL diminishes patients’ QoL and is disfiguring. The lymphatic system has a multifaceted role in immune response and considerable physical function; thus, lymphedema can provoke multiple, acute or chronic, complications. Its management remains a challenging condition for both patients and clinicians with no definite treatment [7, 59]. Studies have shown that the currently evolving surgical treatment with physiological procedures (LVA, VLNT) can combine increased efficacy, low complication rate, and highly positive impact on patient QoL.

Treatment focuses on early intervention to decrease lymphatic load and disease progression. Conservative treatment (i.e. manual lymph drainage/massage/physiotherapy, compression garments, elastic stockings) is widely accepted as the first-line option for lymphedema, and is of foremost importance in the success of physiologic procedures postoperatively. Nevertheless, researchers agree that the earlier LVA or VLNT is performed, the better the outcomes, due to minimal adverse tissue changes in the lymphatic vessels; ablative procedures which resect excessive tissue carry a high risk of complications, including significant scarring, necrosis, and infection [27]. In cases of advanced lymphedema with large amount of excessive tissue that causes obstruction, fibro-lipo-lymph-aspiration with a lymph vessel sparing procedure (FLLA-LVSP procedure) has been proposed as an efficient solution. The duration of the procedure and the recovery time are short, while aesthetic and functional results are immediate without further damage to the lymphatic system [59].

Physiological microsurgical procedures (i.e., LVA, VLNT) reduce excess limb volume, the risk of cellulitis and the need for compression garment use and lymphedema therapy. Prophylactic LVA (LYMPHA) might not be widespread in the day-to-day clinical practice yet, but early results are promising in the primary prevention of BCRL with no risk of undetectable axillary disease [8, 60].

Secondary LVA may result only in temporary improvement of symptoms in advanced stages of lymphedema, thus, leading patients to debulking procedures, such as liposuction for increased limb volume reduction [43]. For this reason, some authors are in favor of CDT in the early stages of lymphedema, while opting out of performing LVA [43]. However, a major advantage over VLNT is that it is a minimally invasive technique, often with practically no complications (i.e. DSL). If LVA is done correctly and symptoms and limb size improvement are observed, CDT must be continued, to preserve those gains in the long term. However, in later stages with more irreversible changes, LVA may not be possible.

In early stages, when no irreversible changes have occurred, the extent of tissue fibrosis is insignificant, and viable lymphatic vessels can be identified, VLNT in a percentage of cases may lead to such limb and symptoms improvements that compression garments and CDT might be discontinued after surgery. Volumetric measurements showed improvement in several studies and in QoL [27, 51]. Another major advantage is that it can be combined with autologous free flap breast reconstruction in a single operation and conditions like neural plexus neuropathy might be treated as well. If there is no improvement in the lymphedema circumference, debulking procedures such as liposuction might follow.

Vascularized lymph vessel transfer (VLVT) is an innovative microsurgical technique that is not as widespread as LVA and VLNT. Its main advantage is that its effectiveness does not depend on the lymphedema’s stage and there is no risk of DSL, in contrast to the other physiological procedures. The surgical technique consists of harvesting only the lymph vessels that are contained in the fat layer, usually from the contralateral limb, thus leaving the lymph nodes intact [61, 62].

### BCRL prevention

The LYMPHA procedure consists of performing LVA at the time of nodal dissection, to prevent postoperative lymphedema in high-risk patients [63]. According to the quantitative meta-analysis by Jørgensen et al., out of the total 176 patients that underwent the same lymphadenectomy procedure, the lymphedema rate in the control group was 56.4% (53/94), while in the experimental group that underwent the prophylactic procedure was 14.6% (12/82) (relative risk 0.33) [8]. Similarly, in Feldman’s et al. study half of patients presented lymphedema, compared to one eighth of patients in the group that received LYMPHA [64]. Hahamoff et al. emphasized the importance of surgical prevention by offering LYMPHA and ALND to 87 women with a 27.5% reduction of the rate of lymphedema in their Institution [65]. Similarly, Ozmen et al. reported a significant decline in the BCRL as a result of simplified LYMPHA (S-LYMPHA) [66].

Another preventing measure for lymphedema, based on the assumption that fibrosis is a main factor of lymphedema, could be the application of a topical dressing, that promotes lymphangiogenesis, erasing or even diminishing fibrosis, as shown in the study of Avraham et al. [41]. A screening test for lymphatic function by ICG lymphography for high-risk patients, such as those undergoing ALND and chemotherapy with docetaxel, may also be useful for detecting patients with subclinical lymphedema, increasing the likelihood of early treatment and cure before volume changes [9, 27].

Subclinical detection of lymphedema using bioimpedance spectroscopy (BIS) may prevent and/or reduce the manifestation of BCRL by early directing treatment modalities [67]. Recent progress with single frequency bioelectrical impedance analysis (SFBIA) allows clinicians to more accurately monitor and/or diagnose BCRL (63.64% sensitivity and 95.15% specificity) [68]. Bioimpedance spectroscopy measures changes in the extracellular fluid, which depending on the L-Dex score lymphedema may be diagnosed [69]. The L-Dex score corresponds to the extracellular ratio of the at-risk or lymphedematous limb to the healthy limb, with a value of + 10 being 3 standard deviations from the healthy mean norm. According to Ridner et al., L-Dex value of ≥ 7 is indicative of clinical lymphedema, while ≥ 6.5 possibly of subclinical lymphedema [70]. Studies show that Shear wave elastography (SWE) is a novel technique for distinguishing early- from advanced-stage lymphedema [71].

In the last few years, the focus has shifted to early intervention, to decrease disease progression and prevent irreversible fibrosis. Vascularized gastroepiploic lymph node transfer (VLNT) is a promising novel technique with good early postoperative outcomes in BCRL reduction [72]. Future work should focus on lymphedema prevention surgery (LPS) by implementing surveillance programs for lymphedema management (SLYM) [73] and technological advancements [56], including robot-assisted supermicrosurgical LVA [74].

### Limitations

There are some limitations in our study. In the literature, many studies have exhibited low quality data with high risk of bias. Moreover, results are often mixed and conflicting with considerable heterogeneity in the study populations; there is a lack of validated objective methods to measure the effects of microsurgical techniques in treating BCRL. These problems have been previously highlighted by several researchers [8, 43].

Furthermore, evaluation and definition of lymphedema must be addressed and standardized as well to facilitate systematic reviews and comparison of results between researchers. The follow-up period should be extended over one year in ALND and over 2 years in SLND, since it is approximately the period when BCRL usually occurs [4, 43]. As for LVA and VLNT, the indications and surgical procedures should be classified and globalized to allow once more results comparison between researchers. Larger series and randomized controlled trials are needed for the investigation of long-term outcomes and the role of prophylactic LVA. As in every surgical intervention, maximizing outcomes while minimizing complications is still dependent on appropriate planning and preparation, skill and training, and meticulous technique and experience [51]. Therefore, each case should be individualized. Lastly, studies show that an integrated multidisciplinary team approach should be established to BCRL treatment as surgical intervention alone does not suffice for a full recovery [12]. Microsurgeons should develop lymphatic surgery programs to facilitate immediate reconstruction [75].

## Conclusion

Together with integrated lymphedema therapy, proper staging, and the appropriate selection of procedure, safe surgical techniques can be used in many patients to treat lymphedema effectively and in a personalized manner [76]. Although the surgical techniques are demanding, thus requiring special equipment, supermicrosurgical training, and personnel, current evidence suggests that outcomes are promising. Future efforts should focus on prevention and early treatment of lymphedema [77].

## Data Availability

Data sharing not applicable to this article as no datasets were generated or analysed during the current study.
